# Persistently high incidence of HIV and poor service uptake in adolescent girls and young women in rural KwaZulu-Natal, South Africa prior to DREAMS

**DOI:** 10.1371/journal.pone.0203193

**Published:** 2018-10-16

**Authors:** Natsayi Chimbindi, Nondumiso Mthiyane, Isolde Birdthistle, Sian Floyd, Nuala McGrath, Deenan Pillay, Janet Seeley, Thembelihle Zuma, Jaco Dreyer, Dickman Gareta, Tinofa Mutevedzi, Justin Fenty, Kobus Herbst, Theresa Smit, Kathy Baisley, Maryam Shahmanesh

**Affiliations:** 1 Africa Health Research Institute, Durban, South Africa; 2 London School of Hygiene and Tropical Medicine, London, United Kingdom; 3 Southampton University, Southampton, United Kingdom; 4 University College London, London, United Kingdom; Institute of Tropical Medicine Antwerp, UNITED STATES

## Abstract

**Background:**

Adolescent girls and young women (AGYW) bear the brunt of the HIV epidemic in South Africa. ‘DREAMS’ aims to reduce HIV incidence through multi-level combination prevention. We describe HIV incidence and uptake of HIV and sexual reproductive health (SRH) by AGYW in KwaZulu-Natal (KZN), prior to DREAMS.

**Methods:**

Longitudinal and cross-sectional analysis of women (15–24 year old) in a population-based HIV incidence cohort within a demographic surveillance site in KZN. Observation time for HIV incidence was person-years at risk while resident. “Current use of contraceptives” and “having an HIV test in the past 12 months” was compared between 2011 and 2015.

**Results:**

In 2015, HIV prevalence was 11.0% and 34.1% and HIV incidence (2011–2015) was 4.54% (95%CI:3.89–5.30) and 7.45% (95%CI:6.51–8.51) per year in 15–19 and 20–24 year olds respectively, with no significant decline compared to 2006–2010. In 2015, 90.7% of 20-24-year-olds were unemployed, 36.4% and 51.7% of 15–19 and 20–24 year olds reported recent migration; 20.9% and 72.6% of 15–19 and 20–24 year olds had ever been pregnant. In 2015, less than 50% reported condom-use at last sex, 15.0% of 15–19 year olds and 48.9% of 20–24 year olds were currently using contraception and 32.0% and 66.7% of 15–19 and 20–24 year olds had tested for HIV in the past 12 months. There had been no improvement compared to 2011. Factors associated with AGYW testing for HIV in the past 12 months were, survey year—2011 more likely than 2015 (aOR = 0.50), number of partners (aOR = 3.25), ever been pregnant (aOR = 2.47) and knowing where to find ART (aOR = 1.54). Factors associated with contraception use were being older (aOR = 4.83); ever been pregnant (aOR = 12.62); knowing where to get ART (aOR = 1.79) and having had an HIV test in past 12 months (aOR = 1.74).

**Conclusion:**

Prior to DREAMS, HIV incidence in AGYW was high. HIV and SRH service uptake did not improve and was suboptimal. Findings highlight the need for combination HIV prevention programmes for AGYW in this economically vulnerable area.

## Introduction

In South Africa six million people are living with HIV, with nearly 400,000 new HIV infections per year. Despite widespread availability of antiretroviral therapy (ART) in South Africa, the risk of acquisition of HIV is particularly high among adolescent girls and young women (AGYW) aged 15–24 years. They are two to four times more likely than their male counterparts to be living with HIV [1; 2; 3].

Young women bear a disproportionate burden of infection because of multiple factors including biology, sexual behaviour, socially constructed gender differences between men and women, as well as differential access to resources and decision-making power [3; 4; 5]. This has led to a growing body of evidence showing the effectiveness of HIV prevention interventions that tackle either biological susceptibility or behavioural risk at an individual level as well as those that tackle structural factors that increase vulnerability to HIV such as education, economic disadvantage, and gender based violence [6; 7; 8]. However, despite the call for combination prevention interventions, by and large these interventions have been provided in silos rather than in combination. [9]

**Fig 1:**
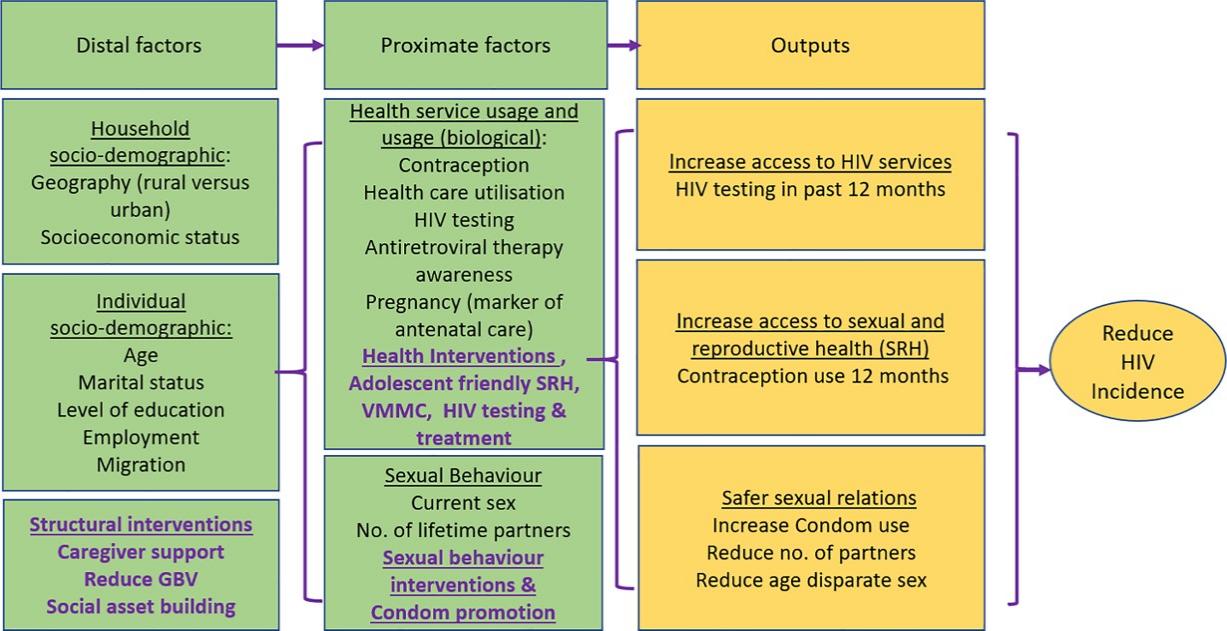
Conceptual framework for effect of combination prevention on HIV incidence in adolescent girls and young women.

The **D**etermined **R**esilient **E**mpowered **A**IDS-free **M**entored **S**afe (DREAMS)’ partnership aims to counter this by introducing an ambitious programme aiming to halt the persistent pattern of HIV infection among young women through providing a combination of evidence-based HIV prevention interventions targeting—biological, behavioural, and structural factors to reduce AGYW vulnerability to HIV [10], with the aim of reducing HIV incidence by 40% by 2017. As part of the Bill & Melinda Gates Foundation (BMGF) funded impact evaluation of DREAMS in a hyper-endemic predominantly rural area of KwaZulu-Natal (KZN), South Africa, we will measure the effect of DREAMS on HIV incidence via the three hypothesised pathways: biological, behavioural, and social protection. The hypothesis that underpins the effectiveness of layering multilevel (structural, behavioural and biological) interventions, on reducing HIV incidence, is in part through promoting **safer sexual relations** and improving the uptake of **HIV testing and Sexual Reproductive Health (SRH) services** by adolescents and young adults (see the simple conceptual framework with DREAMS interventions in purple in Fig 1 below). Our aim in this paper is to describe the trends in HIV incidence and uptake of HIV testing and SRH services in a population-based sample of AGYW prior to DREAMS roll out in 2015. We explore factors associated with service uptake to inform implementation.

## Methods

### Study design and setting

The study drew on existing, longitudinal data collected by Africa Health Research Institute (AHRI) (formally the Africa Centre for Health and Population studies), in the Hlabisa sub-district located in the uMkhanyakude district in northern KwaZulu-Natal–an area for DREAMS investment [11]. The study area is predominately rural and poor compared with other parts of South Africa, with high levels of unemployment [11] and an antenatal HIV prevalence of more than 40% [12]. Since 2000, household-based surveys have been used to collect demographic data from a population of approximately 100,000 individuals living in an area of 438 km^2^ in rural uMkhanyakude District. Households were surveyed 3 times per year, to collect information on birth, deaths and migration patterns for all household members, including non-residents. A resident is a member of a household who normally lives at the same bounded structure as the household. Residents would have spent most of their nights at the bounded structure in the six months since the last round while a non-resident member lives elsewhere for most of the time, and whenever they visit the household at its bounded structure, they always leave again [13]. Resident household members who were aged ≥15 years were invited to participate in an annual face-to-face individual survey, which included an interview on general health and sexual behaviour (which asks questions on pregnancy history, contraceptive use, sexual activity and attitudes to condom use), and collection of a dried blood spot (DBS) for anonymised HIV testing [13]. Teams of two trained fieldworkers (age-sex matched where possible) visited each eligible individual in his or her household and conducted interviews with household members separately to maintain privacy and confidentiality. HIV test results were not routinely given back to participants. Eligibility lists for the individual-level survey were drawn up based on age and residency status in December of the preceding year [11]. The cohort is one of the world’s largest population-based longitudinal HIV surveillance studies, situated at the epi-centre of the dual HIV and TB epidemics. ART first became available in the surveillance area in 2004. In October 2016, South Africa implemented the new WHO ‘treat all’ guidelines, whereby ART is offered to all HIV-positive individuals irrespective of CD4 count.

### Population

The analysis of HIV incidence used household demographic and annual HIV sero-survey data from 2006 to 2015 (the 10 years preceding DREAMS roll out). Individuals were eligible for analysis if they were female, aged 15–24 years, resident in the surveillance area and had participated in the HIV sero-surveillance at least twice during the period 2006–2015, and their first test was HIV negative [14].

For the baseline analysis of HIV prevention and SRH service uptake, all females aged 15–24 years who participated in the general health survey in either 2011 or 2015 were eligible and included. These time points were chosen to include the most recent survey before the introduction of DREAMS in early 2016, and an earlier survey to examine any secular trends in service uptake before the introduction of DREAMS.

### Measurement

#### Outcomes definitions

The two pre-DREAMS measures of uptake of **HIV prevention and SRH services were** explored using questions on: “current use of modern contraceptives”, and “having an HIV test in the past 12 months” (note this can be in any setting but does not include the anonymised testing in the sero-surveillance since those results are not returned to individuals.)

#### Exposure definitions

*Socio-demographic characteristics of the resident household* includes the geographic area and socio-economic status. The socio-economic status variable was constructed using Principal Component Analysis (PCA) based on ownership of household assets and characteristics such as access to piped water, type of toilet, electricity and type of cooking fuel. *Socio-demographic characteristics of AGYW* that were measured were her age, employment (among those that were no longer in school), education level—broken down by those who are still in school and those who have completed school and migration episodes (one migration episode is defined as moving away from the surveillance area and subsequently returning). *Service utilization and awareness* included whether the participant visited a primary health clinic in the past 6 months, whether she had heard about ART and whether she knew where to get ART; and whether she had ever been pregnant. We use ever being pregnant as a surrogate for potential access to antenatal care as the focus on Prevention of Mother to Child Transmission (PMCT) in South Africa has led to good antenatal coverage in uMkhanyakude district where this study is based. A pregnancy cohort from this district found that over 90% of the HIV positive pregnant women were identified and started on ART for PMCT and a drop in Mother to Child transmission rates to 0.5% in 2015 [15]. *Sexual behaviour with male partners* included the number of lifetime partners and whether she had ever had sex or had sex in the past 12 months. Among those who reported having had sex in the past 12 months, the following characteristics were also described: having more than 1 partner in the past 12 months, age- disparate sex (defined as an age difference of 5 years or more with most recent partner), condom use at last sex and condom-less sex (any sex without a condom) in the past 12 months.

### Statistical analyses

All analysis was conducted by STATA version 14 (Stata Corp, College Station, TX, USA). HIV incidence rates were calculated as the number of seroconversions per person-year of observation. Person-time was defined from the date the individual first tested negative in the serosurvey until the latest of date of last negative test or seroconversion date. Periods of non-residency (if individuals moved away from the surveillance area and later returned) were excluded from the calculation of person-time. This was done so to minimise bias towards the population of migrants who return, as we do not have the means to ascertain the HIV status of those who do not return. Seroconversion dates were multiply-imputed (using 250 imputations) as a fraction of the interval between the last negative test date and the first positive test date, assuming uniform distribution. Imputed seroconversion dates which did not fall within a period when the individual was resident in the surveillance area (e.g. if the individual had temporarily moved away) were censored at the latest exit date (the end of the period when the individual was last resident) before the imputed seroconversion date, i.e. the seroconversion event did not contribute to the numerator, and person-time after the residency period did not contribute to the denominator. HIV incidence rates (with 95% confidence intervals (CIs)) were estimated separately for each age group (15–19 years and 20–24 years) and year, and by calendar period (2006–2010 and 2011–2015) to capture trends post-ART roll-out and pre-DREAMS. To assess trends over time, Poisson regression was used to estimate rate ratios (RR) and 95% CIs for the effect of calendar period on incidence. As a sensitivity analysis, periods of non-residency were included in the calculation of incidence (i.e. all seroconversions contributed to the numerator, and person-time during periods of non-residency contributed to the denominator).

Uptake of reproductive and HIV health services were explored using two self-reported outcomes 1) current use of modern contraceptives, and 2) having an HIV test in the past 12 months. Participant characteristics were tabulated for each survey year, stratified by age group. Logistic regression was used to calculate odds ratios (OR) and 95%CIs for the association between socio-demographic, behavioural or health service related and sexual behavioural variables and each of the two outcomes, separately. Robust standard errors were used to account for repeat participation by some participants between survey years. A conceptual hierarchical framework was used to guide the multivariable model building (Fig 1). For each outcome, age and survey year were considered a priori confounders and were included in all models. First, sociodemographic factors whose age- and survey year-adjusted association with the outcome was significant at p<0.10 were included in a multivariable model; those remaining associated at p<0.10 were retained in a core model (Model 1). Next behavioural variables were added to this core model one by one, and retained if they remained significant at p<0.10 (Model 2). Association with health services variables were determined in a similar way. Sexual behavioural variables were added to a model with socio-demographic and behavioural variables (Model 3). Since most of the sexual behaviour questions are only asked of individuals who report having had sex in the past 12 months, the analysis of sexual behaviour was restricted to this sub-group.

### Ethics

Ethical approval for the demographic surveillance study was granted by the Biomedical Research Ethics Committee of the University of KwaZulu-Natal, South Africa, Reference Number BE290/16. Separate informed consent is required for the main household survey, for the individual general health and sexual behaviour questionnaires, and for the HIV sero-survey.

## Results

### Population for the 2011 and 2015 cross sectional analysis

6457 and 6253 AGYW aged 15–24 years were on the eligibility lists for the individual-level surveys in 2011 and 2015, respectively, of whom >90% were contacted. At the time of contact, 1630 (25%) and 1568 (25%) individuals, respectively, were no longer eligible, mostly owing to out-migration. Among those who were contacted and still eligible for the survey, 2864 (67%) AGYW in 2011, and 3154 (72%) in 2015, consented to participation in the general health and/or sexual behaviour survey. AGYW aged 15–19 years were **less** likely to participate than those aged 20–24 (63% vs 73% in 2011; and 68% vs 80% in 2015; p<0.001). In general, participation rates were lower in urban areas than in rural or semi-rural areas.

### Population for the HIV incidence and prevalence analysis

**Fig 2:**
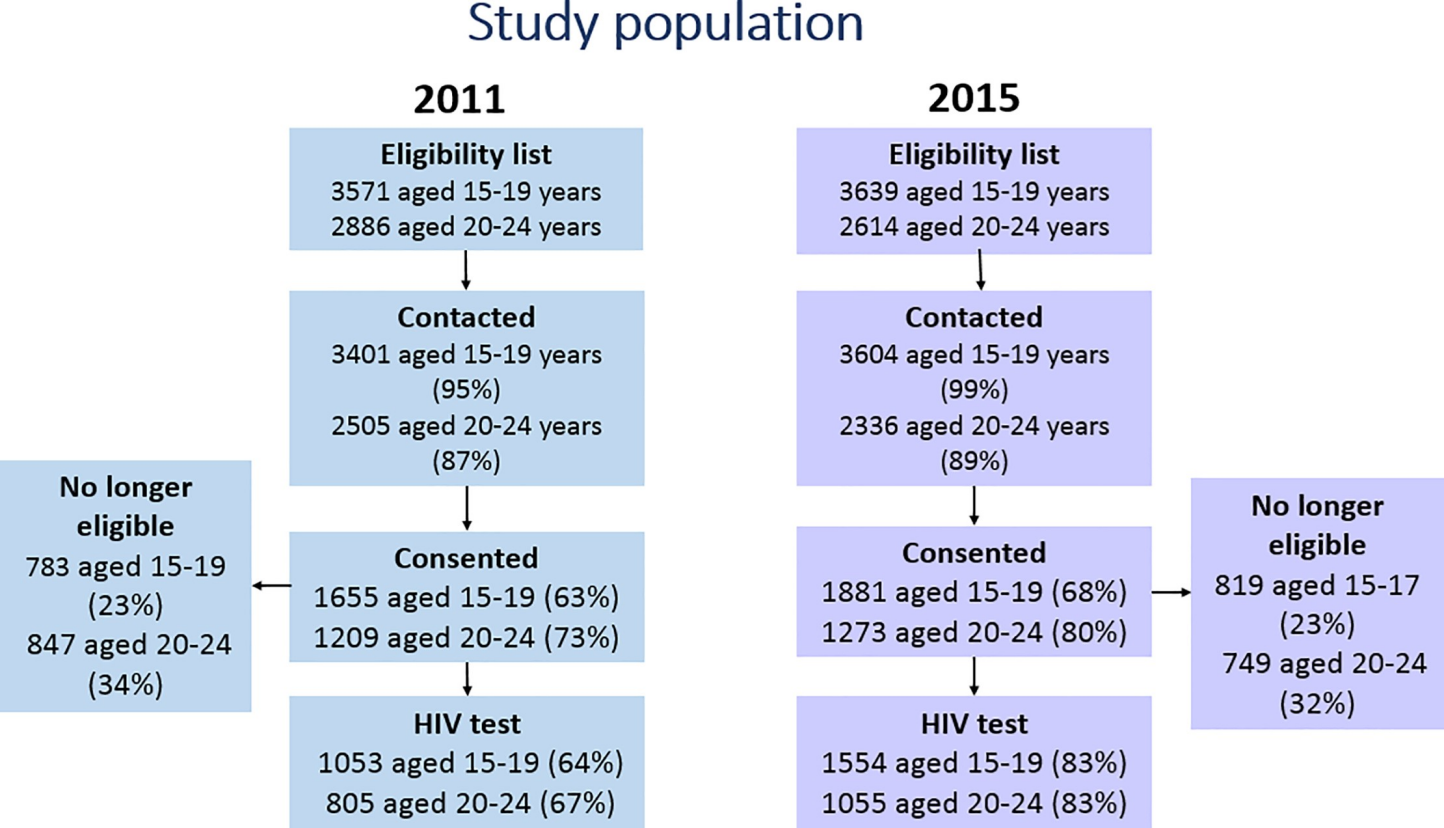
Showing the participation rates of AGYW in the two-time periods.

During 2006–2015, there were 19,730 AGYW aged 15–24 who were ever resident and therefore eligible for at least one of the annual HIV sero-surveys; 85% were eligible in more than one survey round. The majority (>90%) were successfully contacted; at the time of contact, 28%-42% of individuals were no longer eligible, mostly owing to out-migration. Among those who were contacted and still eligible for the survey, participation rates in individual survey years ranged from 37%–58% among AGYW aged 15–19 years, and 29%–51% among AGYW aged 20–24. Overall, 10,628 (65% of 16,343 contacted and still eligible at the time of contact) AGYW ever participated in at least one of the HIV sero-surveys. Of those, 87% were HIV negative at the first HIV test, and 98% were eligible in a subsequent survey round. 6085 contributed data to the HIV incidence analysis (i.e. tested at least twice and were HIV negative at the time of the first test). Of those who consented to the individual behaviour survey participation rates in 2011 and 2015 (for HIV prevalence estimates) were 64% and 83% among AGYW aged 15–19 years, respectively, and 67% and 83% among AGYW aged 20–24 years (see Fig 2)

### Characteristics of study participants

In both survey years, the majority of participants had been members of a household in the survey area since the start of the AHRI demographic surveillance in 2000 (82% in 2011, and 77% in 2015, with no difference between the age groups). The median age of the population across the survey years was the same, 19 years interquartile range (IQR) (17–21) years. In 2011, 67% of participants had out-migrated from the survey area at least once previously and returned; that proportion had reduced to 43% in 2015 (p<0.001). Overall, young women aged 20–24 were more likely to have out-migrated than those aged 15–19 (60% vs 51%).

Table 1 shows a descriptive analysis of the socio-demographic characteristics, health uptake and behavioural factors of the eligible AGYW who participated in the 2011 and 2015 surveys. This is an area of high unemployment among those who are no longer in school which has remained unchanged over the two survey points: 92.2% and 90.7% of 20-24-year-old women were unemployed in 2011 and 2015 respectively. In 2011, 66.8% of 15–19 year olds and 68.3% of 20–24 year olds had out-migrated at least once from the survey area and then returned. In 2015, those proportions had decreased significantly, to 36.4% of 15–19 year olds and 51.7% of 20–24 year olds. Residence of AGYW did not seem to have changed over the two survey periods–most AGYW (60%) lived in rural areas.

**Table 1:** Characteristics of AGYW aged 15–24 by age and year of survey.

Age group	15–19 year			20–24 years		
Year of survey	2011 (N = 1655)	2015 (N = 1881)	p-value	2011 (N = 1209)	2015 (N = 1273)	p-value
**SOCIO-DEMOGRAPHICS**						
**Highest education level**						
*Still in school*			0.01			0.04
Primary	96 (7.4%)	101 (5.5%)		9 (0.9%)	4 (0.3%)	
Some Secondary	920 (71.4%)	1400 (75.6%)		206 (21.5%)	306 (24.6%)	
*Completed school*			0.46			0.01
Primary	23 (1.8%)	21 (1.1%)		47 (4.9%)	32 (2.6%)	
Some Secondary	95 (7.4%)	121 (6.5%)		268 (27.9%)	316 (25.4%)	
Completed secondary/tertiary	155 (12.0%)	210 (11.3%)		430(44.8%)	588(47.2%)	
*Missing*	*366*	*28*		*249*	*27*	
**Marital status**			<0.001			<0.001
Married or engaged	67 (4.1%)	4 (0.2%)		115 (9.7%)	13 (1.1%)	
Single (never married)	1555 (95.9%)	1819 (99.8%)		1065 (90.3%)	1221 (98.9%)	
*Missing*	*33*	*58*		*29*	*39*	
**Employment-(applicable to +18 years)**			0.10			0.11
Not employed	394 (97.8%)	516 (97.7%)		883 (92.2%)	1125 (90.7%)	
Part time	3 (0.7%)	0 (0%)		20 (2.1%)	19 (1.5%)	
Full time	6 (1.4%)	12 (2.2%)		55 (5.7%)	97 (7.8%)	
*Missing*	*316*	*253*		*251*	*32*	
**Socio-economic tertile**			<0.001			<0.001
Low	466 (36.8%)	570 (30.9%)		360 (38.0%)	408 (32.8%)	
Middle	331 (26.1%)	803 (43.5%)		258 (27.2%)	523 (42.0%)	
High	470 (37.1%)	474 (25.7%)		329 (34.7%)	314 (25.2%)	
*Missing*	*388*	*34*		*262*	*28*	
**Geographic area**			0.10			0.03
Peri-Urban	476 (28.8%)	603 (32.1%)		392 (32.4%)	388 (30.5%)	
Rural	1085 (65.6%)	1182 (62.8%)		766 (63.4%)	802 (63%)	
Urban	94 (5.7%)	96 (5.1%)		51 (4.2%)	83 (6.5%)	
**Previous migration episodes**			<0.001			<0.001
None	549 (33.2%)	1196 (63.6%)		383 (31.7%)	615 (48.3%)	
One	890 (53.8%)	547 (29.1%)		569 (47.1%)	472 (37.1%)	
Two or more	216 (13.1%)	138 (7.3%)		257 (21.3%)	186 (14.6%)	
**HEALTH SERVICES USAGE AND BEHAVIOURAL FACTORS**						
**HIV status**			0.10			0.27
Positive	95 (9.0%)	171 (11.0%)		255 (31.7%)	360 (34.1%)	
*Unknown*	602	327		404	218	
**Visited clinic in the past 6 months**			0.15			0.79
Yes	510 (31.5%)	531 (29.2%)		673 (57.0%)	708 (57.5%)	
*Missing*	*35*	*64*		*28*	*42*	
**Ever used contraception**			**0.68**			**0.93**
**Yes**	**273 (16.9%)**	**295 (16.3%)**		**621 (52.8%)**	**643 (52.6%)**	
*Missing*	35	74		32	50	
**Currently using modern contraception**			**0.55**			**0.57**
**Yes**	**255 (15.7%)**	**271 (15.0%)**		**562 (47.7%)**	**598 (48.9%)**	
*Missing*	35	74		32	50	
**HIV test in the past 12 months**		**P<0.001**	**<0.001**			**0.002**
**Yes**	**882 (54.4%)**	**583 (32.0%)**		**852 (72.6%)**	**824 (66.7%)**	
*Missing*	34	58		35	37	
**Heard about ART?**			<0.001			0.001
Yes	1432 (89.6%)	1714 (94.8%)		1127 (96.5%)	1214 (98.5%)	
*Missing*	*57*	*73*		*41*	*41*	
**Know where to get ART?**			<0.001			0.009
Yes	1362 (85.7%)	1698 (94.1%)		1092 (94.1%)	1187 (96.3%)	
*Missing*	*65*	*76*		*48*	*41*	
**Ever been pregnant**			**0.85**			**0.67**
**Yes**	**335 (20.7%)**	**381 (20.9%)**		**861 (73.4%)**	**895 (72.6%)**	
*Missing*	*33*	*60*		*36*	*41*	
**Number of lifetime partners**			**0.001**			**0.006**
**None**	**986 (69.3%)**	**1193 (73.8%)**		**128 (14.4%)**	**172 (20.2%)**	
**One**	**320 (22.5%)**	**340 (21.0%)**		**413 (46.5%)**	**372 (43.8%)**	
**Two or above**	**117 (8.2%)**	**83 (5.1%)**		**347 (39.1%)**	**306 (36.0%)**	
*Missing*	*232*	*265*		*321*	*423*	
**Had sex in the past 12 months**			**0.008**			**0.07**
**Yes**	**426 (29.9%)**	**414 (25.6%)**		**743 (82.5%)**	**672 (79.1%)**	
*Missing*	*231*	*265*		*308*	*423*	
	**AMONG THOSE WHO REPORTED HAVING HAD SEX IN THE PAST 12 MONTHS**					
	**N = 426**	**N = 414**		**N = 743**	**N = 672**	
**More than 1 partner in the past 12 months**			**0.37**			**0.97**
**Yes**	**5 (1.2%)**	**8 (1.9%)**		**9 (1.2%)**	**8 (1.2%)**	
**Age difference of 5+ years with most recent partner**			**0.90**			**0.80**
**Yes**	**86 (20.7%)**	**82 (21.1%)**		**213 (29.7%)**	**185 (29%)**	
*Missing*	*11*	*25*		*25*	*35*	
**Condom use at last sex**			**<0.001**			**0.009**
**No**	**176 (41.3%)**	**227 (55.1%)**		**333 (44.9%)**	**347 (51.8%)**	
*Missing*	*0*	*2*		*1*	*2*	
**Any sex without condom in the past 12 months**			**<0.001**			**0.001**
**Yes**	**271 (63.6%)**	**317 (76.9%)**		**531 (71.6%)**	**530 (79.1%)**	
*Missing*	*0*	*2*		*1*	*2*	

### Pre-DREAMS sexual behaviour and access to HIV and SRH services

Table 1 shows that marriage was uncommon [less than 10% across the survey years] and trends showing a significant decrease among 20–24 year olds, 9.7% in 2011 and 1.1% in 2015. One fifth of 15–19 year olds (20.7% in 2011 and 21.1% in 2015) and about one third of 20–24 year olds (29.7% in 2011 and 29% in 2015) describe age disparate sex with no change over time. A fifth (20.9%) and about three quarters (72.6%) of 15–19 and 20–24 year olds, respectively, had ever been pregnant with no significant difference between the two survey periods. There was no significant change in overall contraception use between 2011 and 2015, with only 16% and 53% reporting contraception use in the two age groups respectively.

Of those who were sexually active and answered the sexual behaviour questions (n = 2466,78%); the proportion of AGYW who reported using a condom at last sex decreased significantly from 58.7% to 44.9% among the 15–19 year olds and from 55.1% to 48.2% among the 20–24 year olds in the two survey periods from 2011 and 2015 respectively. Similarly, more than three quarters of the AGYW reported condom-less sex (any sex without a condom in the past 12 months) in 2015, a significant increase from 2011. While more than a third (39.1% vs 36% in 2011 and 2015 respectively) of 20–24 year olds reported more than one lifetime partner, only 1% reported having more than 1 partner in the past 12 months among this age group (20–24 years) in 2011 and 2015.

The proportion of AGYW reporting testing for HIV in the past 12 months decreased significantly from 2011 to 2015 within the age groups, from 54.4% vs 32% among 15–19 and 72.6% v 66.8% among 20–24 year olds respectively. Health service uptake was low in both survey years, slightly lower in 2015 than in 2011, especially among the 15–19 year olds with less than a third (29.2%) and 57.5% 20–24 year olds in 2015, reporting visiting a clinic in the past 6 months. However, the majority of the AGYW 15–19 (94.8%) and 20–24 years olds (98.5%) in 2015 knew where to get ART—there was a significant increase from 2011 in both age groups, 89.7% and 96.5% respectively.

### HIV prevalence and incidence

HIV prevalence increased but not significantly over the two survey years, from 9.0% (95%CI = 7.4‒10.9%) in 2011 to 11.0% (95%CI = 9.5‒12.7%) in 2015 among AGYW aged 15–19 years, and 31.7% (95%CI = 28.5‒35.0%) to 34.1% (95%CI = 31.3‒37.1%) among 20–24 year olds (Table 2). HIV incidence during 2011‒2015 was 4.7% (95% CI 3.9–5.3) per 100 person-years in AGYW aged in 15–19 and 7.5% (95% CI 6.5–8.5) in those aged 20–24. Although incidence in both age groups was slightly lower in 2011‒2015 than during 2006–2010, there was no evidence of a significant difference in the rate of new infections between the two calendar periods. However, there was weak evidence of a decreasing trend in annual incidence among AGYW aged 15–20 years from 2011 to 2015 (RR for linear trend from one year to the next = 0.93, 95%CI = 0.87–1.00, p = 0.06; S1 Table and S1 Fig). In the sensitivity analysis including periods of non-residency, the estimates of incidence were very similar and the conclusions regarding change over time were the same (S2 Table).

**Table 2:** HIV incidence estimates in AGYW aged 15–24 years, by age group and calendar period.

Age group	Calendar period	New HIV infections	Person-years	Incidence rate / 100 person-years	Rate ratio (95% CI) ^1^
15–19 years	2006–2010	254	5395	4.71 (4.10–5.41)	1
	2011–2015	197	4330	4.54 (3.89–5.30)	0.97 (0.78–1.19)
20–24 years	2006–2010	340	4462	7.62 (6.71–8.65)	1
	2011–2015	289	3881	7.45 (6.51–8.51)	0.98 (0.81–1.18)

^1^Rate ratio comparing HIV incidence in the period 2011–2015 to that in 2006–2015, adjusted for current age.

### AGYW factors associated with HIV testing in the past 12 months

Table 3 shows the AGYW’s factors associated with HIV testing in the past 12 months. After adjustment, AGYW in 2015 were less likely to have tested for HIV than in 2011 (aOR = 0.50, 95%CI 0.44–0.56). Being older (aOR = 2.87, 95%CI 2.53–3.25) and increasing levels of education (aOR = 2.16, 95% CI1.64–2.84) were both associated with HIV test in the past 12 months.

**Table 3:** Factors associated with HIV testing in the past 12 months.

	n testing for HIV/ N (%)	Crude OR (95% CI)	Adjusted OR (95% CI)^1^
**Sociodemographic factors**	**Adjusted OR are adjusted for age, year and education**		
**Year**		P<0.001	P<0.001
2011	1734 / 2795 (62.0%)	1	1
2015	1407 / 3059 (46.0%)	0.52 (0.47–0.58)	0.50 (0.44–0.56)
**Age group**		P<0.001	P<0.001
15–19	1465 / 3444 (42.5%)	1	1
20–24	1676 / 2410 (69.5%)	3.08 (2.77–3.44)	2.87 (2.53–3.25)
**Geographic area**		P = 0.63	P = 0.39
Rural	2007 / 3721 (53.9%)	1	1
Peri-urban	973 / 1818 (53.5%)	0.98 (0.88–1.10)	0.94 (0.83–1.07)
Urban	161 / 315 (51.1%)	0.89 (0.71–1.13)	0.86 (0.66–1.12)
**Marital status**		P<0.001	P = 0.22
Married	139 / 196 (70.9%)	1	1
Single	2983 / 5628 (53.0%)	0.46 (0.34–0.63)	0.78 (0.53–1.16)
**Education**		P< 0.001	P<0.001
Primary	135 / 324 (41.7%)	1	1
Some secondary	1677 / 3525 (47.6%)	1.27 (1.01–1.60)	1.38 (1.07–1.77)
Completed secondary/above	925 / 1356 (68.2%)	3.00 (2.34–3.86)	2.16 (1.64–2.84)
**Employment**		P = 0.03	P = 0.49
Not employed	1834 / 2852 (64.3%)	1	1
Employed	149 / 208 (71.6%)	1.40 (1.03–1.91)	1.12 (0.81–1.55)
**SES**		P = 0.36	P = 0.58
Low	919 / 1745 (52.7%)	1	1
Middle	962 / 1870 (51.4%)	0.95 (0.84–1.09)	1.06 (0.92–1.22)
High	836 / 1551 (53.9%)	1.05 (0.92–1.21)	0.99 (0.85–1.14)
**Previous migration episodes**		P<0.001	P = 0.32
None	1301 / 2664 (48.8%)	1	1
One	1351 / 2413 (56.0%)	1.33 (1.19–1.49)	1.01 (0.89–1.15)
Two or more	489 / 777 (62.9%)	1.78 (1.51–2.10)	1.16 (0.95–1.41)
**Behavioural factors**	**Adjusted Or are adjusted for age, year, education, lifetime partners, ever pregnant, and knowing were to get ART.**		
**Number of lifetime partners**		P<0.001	P<0.001
None	726 / 2467 (29.4%)	1	1
One	1018 / 1437 (70.8%)	5.83 (5.05–6.73)	3.00 (2.39–3.77)
Two or more	642 / 848 (75.7%)	7.47 (6.23–8.96)	3.25 (2.43–4.35)
**Ever been pregnant**		P<0.001	P<0.001
No	1222 / 3356 (36.4%)	1	1
Yes	1892 / 2457 (77.0%)	5.85 (5.20–6.58)	2.47 (1.94–3.15)
**Currently using contraception**		P<0.001	P = 0.507
No	1819 / 4113 (44.2%)	1	1
Yes	1283 / 1679 (76.4%)	4.09 (3.59–4.65)	1.08 (0.87–1.33)
**Sex in the past 12 months**		P<0.001	P<0.001
No	768 / 2523 (30.4%)	1	1
Yes	1626 / 2243 (72.5%)	6.02 (5.30–6.84)	2.80 (2.26–3.46)
**Know where to get ART**		P<0.001	P = 0.001
No	162 / 449 (36.1%)	1	1
Yes	2952 / 5337 (55.3%)	2.19 (1.80–2.68)	1.54 (1.19–2.00)
**Sexual behaviour among those reporting sex in past 12m**	**Adjusted OR are adjusted for age, year, education, ever pregnant, knowing were to get ART, and condom use at last sex.**		
**>1 partner in past 12m**		P = 0.76	P = 0.84
No	1605 / 2213 (72.5%)	1	1
Yes	21 / 30 (70.0%)	0.88 (0.40–1.94)	1.10 (0.44–2.77)
**Most recent partner >5y older**		P = 0.33	P = 0.29
No	1146 / 1591 (72.0%)	1	1
Yes	414 / 558 (74.2%)	1.12 (0.89–1.39)	1.14 (0.89–1.46)
**Used condom at last sex**		P = 0.006	P = 0.005
No	753 / 1078 (69.9%)	1	1
Yes	871 / 1160 (75.1%)	1.30 (1.08–1.57)	1.34 (1.09–1.65)
**Condomless sex in past 12m**		P = 0.58	P = 0.07
Yes	1196 / 1641 (72.9%)	1	1
No	428 / 597 (71.7%)	0.95 (0.78–1.18)	0.77 (0.57–1.03)
**Casual partner in past 12m**		P = 0.12	P = 0.52
No	1522 / 2088 (72.9%)	1	1
Yes	104 / 155 (67.1%)	0.76 (0.54–1.07)	0.88 (0.61–1.28)

AGYW with a greater number of lifetime partners (aOR = 3.25, 95% CI 2.43–4.35); those who had ever been pregnant (aOR = 2.47, 95%CI 1.94–3.15), and those who knew where to get ART (aOR = 1.54, 95%CI 1.19–2.00) were more likely to test for HIV in the past 12 months. Sexually active AGYW who self-reported condom use at last sex in the past 12 months were more likely to test for HIV than those who did not report condom use (aOR = 1.34, 95%CI 1.09–1.65). However, having any condom-less sex in the past 12 months, having a casual partner in the past 12 months and having more than one partner in the past 12 months were not associated with HIV testing in the last 12 months.

### AGYW factors associated with current use of modern contraception

Table 4 shows that in the adjusted analysis, factors associated with current use of contraception among AGYW were older age group (aOR = 4.83, 95% CI 4.18–5.57); and completed secondary education (aOR = 1.59, 95%CI 1.15–2.22). AGYW in the highest socio-economic status were less likely to report contraception use.

**Table 4:** Factors associated with current use of modern contraception.

	n using contraception/ N (%)	Crude OR (95% CI)	Adjusted OR (95% CI)
**Sociodemographic factors**	**Adjusted OR are adjusted for age, year, education and SES.**		
**Year**		P = 0.66	P = 0.82
2011	817 / 2797 (29.2%)	1	1
2015	869 / 3030 (28.7%)	0.97 (0.87–1.09)	0.98 (0.86–1.13)
**Age group**		P<0.001	P<0.001
15–19	526 / 3427 (15.3%)	1	1
20–24	1160 / 2400 (48.3%)	5.16 (4.57–5.82)	4.83 (4.18–5.57)
**Geographic area**		P = 0.19	P = 0.13
Rural	1042 / 3706 (28.1%)	1	1
Peri-urban	550 / 1805 (30.5%)	1.12 (0.99–1.27)	1.14 (0.97–1.32)
Urban	94 / 316 (29.7%)	1.08 (0.85–1.38)	1.25 (0.94–1.67)
**Marital status**		P<0.001	P = 0.22
Married	81 / 197 (41.1%)	1	1
Single	1604 / 5627 (28.5%)	0.57 (0.43–0.76)	0.78 (0.53–1.16)
**Education**		P<0.001	P = 0.001
Primary	68 / 317 (21.5%)	1	1
Some secondary	848 / 3510 (24.2%)	1.17 (0.88–1.54)	1.24 (0.91–1.69)
Completed secondary/above	569 / 1349 (42.2%)	2.67 (2.00–3.57)	1.59 (1.15–2.22)
**Employment**		P = 0.12	P = 0.62
Not employed	1182 / 2842 (41.6%)	1	1
Employed	98 / 208 (47.1%)	1.25 (0.95–1.65)	1.08 (0.81–1.44)
**SES**		P = 0.01	P = 0.002
Low	527 / 1726 (30.5%)	1	1
Middle	550 / 1862 (29.5%)	0.95 (0.83–1.10)	0.96 (0.82–1.12)
High	404 / 1551 (26.0%)	0.80 (0.69–0.93)	0.76 (0.64–0.89)
**Previous migration episodes**		P<0.001	P = 0.09
None	724 / 2644 (27.4%)	1	1
One	684 / 2412 (28.4%)	1.05 (0.93–1.19)	0.93 (0.80–1.08)
Two or more	278 / 771 (36.1%)	1.50 (1.26–1.77)	1.17 (0.95–1.44)
**Behavioural factors**	**Adjusted OR are adjusted for age, year, education, SES, ever pregnant, testing for HIV in past 12m, and knowing were to get ART.**		
**Ever been pregnant**		P<0.001	P<0.001
No	227 / 3360 (6.8%)	1	1
Yes	1450 / 2454 (59.1%)	19.93 (17.05–23.30)	12.62 (10.29–15.47)
**Sex in the past 12 months**^**1**^			
No	16 / 2526 (0.6%)	–	–
Yes	1262 / 2242 (53.3%)	–	–
**Tested for HIV in past 12m**		P<0.001	P<0.001
No	396 / 2690 (14.7%)	1	1
Yes	1283 / 3102 (41.4%)	4.09 (3.59–4.65)	1.74 (1.47–2.07)
**Know where to get ART**		P<0.001	P<0.001
No	46 / 443 (10.4%)	1	1
Yes	1620 / 5283 (30.7%)	3.82 (2.80–5.20)	2.68 (1.79–4.03)
**Sexual behaviour among those reporting sex in past 12m**	**Adjusted OR are adjusted for age, year, education, SES, ever pregnant, testing for HIV in past 12m, knowing were to get ART, condom use at last sex, and any condomless sex in past 12m.**		
**>1 partner in past 12m**		P = 0.68	P = 0.62
No	1244 / 2212 (56.2%)	1	1
Yes	18 / 30 (60.0%)	1.17 (0.56–2.44)	1.27 (0.50–3.19)
**Most recent partner >5y older**		P = 0.52	P = 0.54
No	892 / 1589 (56.1%)	1	1
Yes	322 / 558 (57.7%)	1.07 (0.88–1.29)	1.07 (0.86–1.34)
**Used condom at last sex**		P<0.001	P<0.001
No	510 / 1078 (47.3%)	1	1
Yes	750 / 1159 (64.7%)	2.04 (1.73–2.42)	1.82 (1.44–2.30)
**Condomless sex in past 12m**		P<0.001	P = 0.01
Yes	867 / 1641 (52.8%)	1	1
No	393 / 596 (65.9%)	1.73 (1.42–2.10)	1.45 (1.09–1.92)
**Casual partner in past 12m**		P = 0.45	P = 0.15
No	1172 / 2090 (56.1%)	1	1
Yes	90 / 152 (59.2%)	1.14 (0.81–1.59)	1.31 (0.91–1.90)

^1^Frequency of current contraception use is shown for information, but ORs not estimated because of very small number who report using contraception in group with no sex in past 12 months.

The behavioural factors associated with current contraception use were ever being pregnant (aOR = 12.62, 95% CI 10.29–15.47), having had an HIV test in the past 12 months (aOR = 1.74, 95% CI 1.47–2.07); knowing where to get ART (aOR = 2.68, 95% CI 1.79–4.03). Sexually active AGYW who used a condom at last sex (aOR = 1.82, 95%CI 1.44–2.30), were more likely to also be using modern contraception and less likely to report condom-less sex in the past 12 months.

## Discussion

Our findings indicate that in the 10 years prior to DREAMS roll out in this area of KwaZulu-Natal, rates of new HIV infections among AGYW aged 15–24 were extremely high with no statistically significant evidence of decline. Despite high levels of awareness of where to receive antiretroviral treatment, uptake of available HIV and reproductive health services was low and had not increased over the past 5 years and several indicators, such as consistent condom use and uptake of HIV testing, had worsened. Less than one in three 15–19 year olds had tested for HIV in the past 12 months, or visited a clinic in the past six months. Whilst, 20-24-year-olds reports of visiting clinics and HIV testing was associated with their self-reported pregnancies, suggesting that the HIV testing and clinics visits were related to antenatal care in this setting with excellent prevention of mother to child transmission of HIV (PMCT) programme [15].

The likelihood of HIV testing seems to have halved between the two time points of 2011 and 2015 and are lower than rates reported elsewhere among young people in South Africa [16]. Similarly, only one in ten 15–19 year olds and less than half of the 20–24 year olds were currently using modern contraception, with one fifth of adolescents having ever been pregnant and pregnancy being common by the age of 24. Worryingly, less than half of those who self-reported being sexually active were reporting the use of condoms at last sex and more than three quarters reported condom-less sex over the past year, with suggestions that self-reported condom use is decreasing.

Our findings also confirm the socio-economic challenges that AGYW living in this deprived area of northern KZN face, with 9 in 10 having no employment (this includes part-time employment) and nearly 2 out of 3 of those aged 20–24 reporting at least one recent migration episode. Separate work that we have conducted [17] suggests that the community are aware that urban environments in this setting act as transition points for young adults seeking employment. These areas are perceived to be riskier environments, not least because of the loss of the community safety networks that adolescents and young adults can rely on in the more rural areas [17]. Similar, to other studies from South Africa and elsewhere in sub-Saharan Africa, we found that having secondary education was associated with higher contraception use and greater awareness of HIV status [18] and being in school was found to be protective of AGYW [19]. This supports the emphasis in the DREAMS initiative around supporting young girls to stay in school, but also raises the need for social protection during transitions from school to employment and into areas which are on the route of migration.

The strength of association between ever being pregnant and both HIV testing and contraception use, suggests that HIV testing and contraception are primarily taken up by AGYW after the first episode of pregnancy in this rural setting. Further, we found a positive association between current use of contraception and knowledge of where to get ART—a proxy measure of health-related knowledge of access to services. This and the poor service usage in younger girls and those who are not yet pregnant may in part reflect the success of the PMTCT programme in reaching women once they become pregnant [15]. These finding are in keeping with a study in rural Western Cape that also found that having one or more children was associated with increased contraception [20]. Similarly, in a national survey, levels of knowledge among women aged 15–19 years were lower than those of older women for each contraceptive method particularly intrauterine devices (IUD) and emergency contraception [21]. All suggesting that knowledge and access to contraceptives increases through antenatal and perinatal care and age.

These findings also suggest that the part of the bottleneck may be the provision of HIV testing and reproductive health services through already busy primary health care clinics [22] which young people and health care professionals perceive as a place for the very sick or to support maternal and child healthcare, with little space or sympathy for the well looking adolescent and young adult [23]. A study with young girls aged 14–20 years found that barriers to contraception use included pressure from male partners and family members to have a baby or prove their fertility and fear that contraception causes infertility–both from the healthcare providers and religious figures. Nurses’ unwillingness to acknowledge adolescents’ concerns as contraceptive users (for example absence of menstruation), also undermined the effective use of contraception by girls [24]. These perceptions combined with long waiting times or cost of travel may explain the poor healthcare usage prior to first pregnancy. Strengthening the implementation of the SA guidance for adolescent friendly services, through DREAMS may improve access to local services for AGYW [10].

These data suggest that those women who have higher perception of risk (i.e. those with higher partner numbers) or perceive their partner to be higher risk (i.e. those that report condom use at their last sex) were more likely to have tested for HIV. However, the overall limited condom use coupled with the lack of association between condom-less sex and having an HIV test, suggest that there is limited awareness of the risk of any unprotected sexual intercourse in this hyper-endemic setting. This and the high levels of pregnancy by the age of 24 suggests the need for more nuanced messaging around sexual risk, which goes beyond simplistic messaging around number of partners and begins to integrate HIV prevention within the wider discourse around protecting fertility, safe conception and good sexual health. The provision of HIV testing for both AGYW and their male partners, integrating HIV testing messaging with those of better sexual health and healthy conception, offer of condoms and Pre-Exposure Prophylaxis as part of the sexual health package, and scaling up accessible and adolescent friendly HIV testing as part of comprehensive sexual and reproductive health that reach AGYW before they first attend the primary healthcare clinic for antenatal care, are all areas that need strengthening to reduce the biological susceptibility to HIV [10].

Strengths of our study include a large population-based survey, with detailed interviews conducted annually and an annual HIV sero-survey. This allowed us to examine trends over time in HIV incidence and in uptake of available HIV services. As we used existing surveillance data to examine the association between uptake of services (HIV testing and contraception) and different exposures, we are limited by the exposures and outcomes that were measured consistently over time and could not include exposures that were not measured. The strength of this is that the data has been collected in a similar way for over a decade and therefore lends itself to comparison. The limitation is that we did not have measures of some exposures, such as gender based violence, alcohol use and transactional sex that may be important drivers of the outcome. Further limitations of the study include that HIV testing, contraception use as well as sexual behaviours, were self-reported in a face-to-face interview, thus may be subject to social desirability bias. Participation rates in any one annual survey are relatively low, with approximately a third of individuals no longer being eligible at the time of the visit, mostly as a result of having out-migrated. As a result of the limitations cited above, the overall levels of self-reported HIV testing and number of sexual partners in the past 12 months may be over and under-reported respectively due to non-response and social desirability biases. Individuals who choose not to respond to sexual health questions may be different from those who respond with regards to sexual or health seeking behaviour, and participants who respond may feel pressured to provide positive answers for sensitive topics like HIV.

Our method of calculating HIV incidence censors imputed seroconversion dates that do not occur during a residency episode, and excludes periods of non-residency from the calculation of person-time at risk. This provides a measure of new HIV infections among individuals who are resident in the surveillance area, and avoids introducing bias by including those migrants who return/survive long enough to participate in the HIV surveillance again. However, this is a highly mobile population, and therefore excluding non-residents may not give a complete picture of HIV incidence in this area. Reassuringly, when we include the non-residents in the analysis there is very little difference in the estimates of HIV incidence (S1 Table). As noted the data for this study was collected through an ongoing demographic surveillance that is being used as baseline data for the DREAMS evaluation. Questions on sexual behaviour (now self-filled by participant) and service use have been strengthened in the DREAMS evaluation to be able to show change in social or behavioural findings in the evaluation.

In summary, we found a very high incidence of HIV among AGYW aged 15–24 years, with little evidence of decline over the past 10 years. Uptake of HIV testing and SRH services was low, whilst condom-less sex remains high, with no increases in HIV testing, contraception uptake or condom use over the past five years. Structural drivers of HIV and poor health are prevalent in this setting and remain associated with poorer service uptake. This supports the urgent need to scale-up multi-level HIV prevention, as planned through DREAMS for AGYW in this rural and economically vulnerable area. However, poor awareness of risk, lack of support to navigate complex multi-level prevention, and barriers to providing accessible and friendly HIV and sexual health services for AGYW and young men through existing primary care services, may still limit the effectiveness of DREAMS. This evidence suggests that for hyper-endemic rural settings such as ours, we may need to consider innovative models of community-based sexual health programs, co-developed with young people and integrating HIV Pre-Exposure Prophylaxis to protect this generation of AGYW at very high risk of HIV acquisition and pregnancy.

## Supporting information

S1 Table HIV incidence estimates in AGYW aged 15–24 years by age group and year, 2006–2016. (DOCX)

S2 Table HIV incidence estimates in AGYW aged 15–24 years, by age group and calendar period (including periods of non-residency). (DOCX)

S1 Fig HIV incidence in AGYW. (DOCX)
