# Supplementary material for: Persistently high incidence of HIV and poor service uptake in adolescent girls and young women in rural KwaZulu-Natal, South Africa prior to DREAMS
Source: PLoS One. 2018 Oct 16;13(10):e0203193. doi: 10.1371/journal.pone.0203193 (PMC6191091; doi:10.1371/journal.pone.0203193)
Supplement: S1 Table — (DOCX) [file pone.0203193.s001.docx]

S1 Table. HIV incidence estimates in AGYW aged 15–24 years by age group and year, 2006–2016

| **Age group** | **Year** | **New HIV infections** | **Person-years** | **Incidence rate / 100 person-years** | **Rate ratio (95% CI) (reference 2006)** | **Linear rate ratio (95% CI) ^1^** |
| --- | --- | --- | --- | --- | --- | --- |
| **15–19 y** | 2006 | 58 | 1279 | 4.53 (3.28 -6.26 ) | 1 |  |
|  | 2007 | 56 | 1202 | 4.60 (3.31 -6.41 ) | 1.02 (0.63 -1.65 ) | 1.01 (0.91-1.13) |
|  | 2008 | 51 | 1136 | 4.43 (3.08 -6.37 ) | 0.98 (0.60 -1.60 ) | P=0.82 |
|  | 2009 | 49 | 945 | 5.15 (3.55 -7.48 ) | 1.14 (0.69 -1.88 ) |  |
|  | 2010 | 41 | 832 | 4.84 (3.18 -7.36 ) | 1.07 (0.63 -1.81 ) |  |
|  | 2011 | 42 | 878 | 4.78 (3.23 -7.07 ) | 1.05 (0.63 -1.75 ) |  |
|  | 2012 | 42 | 777 | 5.35 (3.67 -7.80 ) | 1.18 (0.72 -1.95 ) | 0.93 (0.87-1.00) |
|  | 2013 | 44 | 860 | 5.07 (3.50 -7.35 ) | 1.12 (0.69 -1.83 ) | P=0.06 |
|  | 2014 | 38 | 929 | 4.11 (2.77 -6.10 ) | 0.91 (0.55 -1.51 ) |  |
|  | 2015 | 30 | 885 | 3.39 (2.21 -5.20 ) | 0.75 (0.44 -1.28 ) |  |
| **20–24 y** | 2006 | 67 | 822 | 8.10 (5.99 -10.94) | 1 |  |
|  | 2007 | 67 | 879 | 7.64 (5.61 -10.39) | 0.94 (0.60 -1.48 ) | 0.98 (0.89-1.08) |
|  | 2008 | 68 | 916 | 7.44 (5.42 -10.20) | 0.92 (0.59 -1.43 ) | P=0.65 |
|  | 2009 | 70 | 928 | 7.51 (5.53 -10.21) | 0.93 (0.60 -1.43 ) |  |
|  | 2010 | 67 | 918 | 7.31 (5.39 -9.93 ) | 0.90 (0.59 -1.39 ) |  |
|  | 2011 | 60 | 859 | 6.94 (4.94 -9.75 ) | 0.86 (0.54 -1.36 ) |  |
|  | 2012 | 61 | 823 | 7.44 (5.46 -10.14) | 0.92 (0.60 -1.41 ) | 1.01 (0.95-1.08) |
|  | 2013 | 59 | 783 | 7.49 (5.41 -10.36) | 0.92 (0.60 -1.43 ) | P=0.68 |
|  | 2014 | 62 | 753 | 8.25 (5.96 -11.41) | 1.02 (0.65 -1.60 ) |  |
|  | 2015 | 46 | 663 | 6.94 (4.79 -10.05) | 0.86 (0.53 -1.38 ) |  |

^1^Rate ratio for linear trend in HIV incidence from one year to the next, between 2006-2010 (unshaded area) and 2011-2015 (shaded area), adjusted for current age.
