# Supplementary material for: Persistently high incidence of HIV and poor service uptake in adolescent girls and young women in rural KwaZulu-Natal, South Africa prior to DREAMS
Source: PLoS One. 2018 Oct 16;13(10):e0203193. doi: 10.1371/journal.pone.0203193 (PMC6191091; doi:10.1371/journal.pone.0203193)
Supplement: S2 Table — (DOCX) [file pone.0203193.s002.docx]

S2 Table. HIV incidence estimates in AGYW aged 15–24 years, by age group and calendar period (including periods of non-residency)

| **Age group** | **Calendar period** | **New HIV infections** | **Person-years** | **Incidence rate / 100 person-years** | **Rate ratio  (95% CI) ^1^** |
| --- | --- | --- | --- | --- | --- |
| 15–19 years | 2006–2010 | 272 | 5744 | 4.73 (4.13 -5.41 ) | 1 |
|  | 2011–2015 | 205 | 4449 | 4.60 (3.96 -5.35 ) | 0.98 (0.80 -1.20 ) |
| 20–24 years | 2006–2010 | 382 | 5061 | 7.54 (6.71 -8.47 ) | 1 |
|  | 2011–2015 | 318 | 4314 | 7.37 (6.50 -8.35 ) | 0.97 (0.82 -1.16 ) |

^1^Rate ratio comparing HIV incidence in the period 2011-2015 to that in 2006-2015, adjusted for current age
