# Supplementary material for: Persistently high incidence of HIV and poor service uptake in adolescent girls and young women in rural KwaZulu-Natal, South Africa prior to DREAMS
Source: PLoS One. 2018 Oct 16;13(10):e0203193. doi: 10.1371/journal.pone.0203193 (PMC6191091; doi:10.1371/journal.pone.0203193)
Supplement: S1 Fig — (DOCX) [file pone.0203193.s003.docx]

S1 Fig. HIV incidence in AGYW
